# Complete Genome Sequence of Bacillus badius NBPM-293, a Plant Growth-Promoting Strain Isolated from Rhizosphere Soil

**DOI:** 10.1128/MRA.00977-21

**Published:** 2021-11-11

**Authors:** Lei Zhu, Yueying Wang, Jiancheng Lu, Simeng Liu, Yong Min, Xiaoyan Liu, Yimin Qiu, Hongtao Hu, Ronghua Zhou

**Affiliations:** a National Biopesticide Engineering Technology Research Center, Hubei Biopesticide Engineering Research Center, Hubei Academy of Agricultural Sciences, Biopesticide Branch of Hubei Innovation Centre of Agricultural Science and Technology, Wuhan, People’s Republic of China; University of Maryland School of Medicine

## Abstract

*Bacillus* species have a long history of widespread use in biocontrol and crop growth-promoting fields. Here, we present the genome sequence of the rhizobacterium *B. badius* NBPM-293. The genome sequence will provide valuable information for a better understanding of the mechanism of plant growth promotion.

## ANNOUNCEMENT

*Bacillus* agents have been widely used around the world for biocontrol since the last century, with the enormous advantage of providing stable and long-living bioformulations (1). This study aimed to isolate plant growth-promoting rhizobacteria from rhizosphere soil. Using sterilized double distilled water, rhizosphere soil was washed off a healthy Fengtou ginger root, collected in an area of endemicity of ginger wilt disease in Laifeng County, China. The bacterial suspension was 10-fold serial diluted, spread onto Luria-Bertani (LB) medium plates, and incubated at 30°C overnight. Then, strain NBPM-293 was obtained from 553 different single colonies. To identify the strain, PCR of the 16S rRNA gene was performed using the universal primers 27F (5′-AGAGTTTGATCCTGGCTCAG-3′) and 1492R (5′-TACGGCTACCTTGTACGACTT-3′). Using the EzBioCloud database (2), phylogenetic analysis of the 16S rRNA gene identified NBPM-293 as belonging to Bacillus badius (99.86% identity to the 16S rRNA gene of *B. badius* MTCC 1458^T^ [GenBank accession number JXLP01000009.1]).

To investigate the mechanisms of plant growth promotion, the genome of NBPM-293 was sequenced. Genomic DNA was extracted using the modified SDS lysis method from an overnight culture in LB medium at 30°C, 220 rpm, and purified using an Omega column and Beckman AMPure XP beads. The DNA purity, concentration, and integrity were examined using a NanoDrop One spectrophotometer (NanoDrop Technologies, Wilmington, DE, USA), Qubit 3.0 fluorometer (Life Technologies, Carlsbad, CA, USA), and 1% agarose gel electrophoresis, respectively. For short-read sequencing, DNA was sheared to 350 bp using a Covaris LE220 focused ultrasonicator (USA) for library construction. The quality of the DNA fragments was checked using a Bioanalyzer 2100 system (Agilent Technologies, USA). A paired-end DNA library was prepared using a NEBNext Ultra II DNA library prep kit for Illumina (NEB, USA), and sequencing was performed on an Illumina NovaSeq 6000 instrument, using 150-nucleotide (nt) reads. The adapter sequences were removed and low-quality reads were filtered using SOAPnuke v.2.1.2 (3) with default settings, except that the filter threshold was 10. In total, 7,245,346 high-quality reads (1,086,801,900 bp) were obtained with an average coverage of 243.5×. For long-read sequencing, using the same genomic DNA preparation, sequencing was performed on the GridION Nanopore sequencing platform by Oxford Nanopore Technologies (ONT). Genomic DNA was purified and directly constructed into a library using a ligation sequencing kit (SQK-LSK109). The DNA library was barcoded following the ONT standard protocol using the native barcoding expansion 1-12 (PCR-free) kit (EXP-NBD104), loaded onto R9.4 flow cells and run on a ONT GridION device. For the raw sequences, base-calling, barcode segmentation, and adapter sequence removal were performed using Guppy v.4.4.2 (4) with default settings. Finally, 30,025 reads (1,000,023,354 bp) were obtained with an average coverage of 236.8×. The average ONT read length was 33,306.4 bp and the *N*_50_ value was 37,255 bp. The high-quality short-read and long-read sequences were assembled into a complete chromosome and plasmids using Unicycler v.0.4.9 (5) with default settings.

The genome size of NBPM-293 is 4,289,048 bp, containing a circular chromosome and six plasmids (Table 1). The genome sequence was annotated using NCBI PGAP v.5.3 (6) with default settings. A clustered regularly interspaced short palindromic repeat (CRISPR) was predicted using CRISPRCasFinder v.4.2.2 (7) with default settings. The annotation showed that the genome of NBPM-293 contains 4,374 genes without any CRISPR structure (Table 1).

**TABLE 1:** Genome features of Bacillus badius NBPM-293

Genetic element	Length (bp)	G+C content (%)	No. of genes	No. of tRNAs	No. of rRNAs	GenBank accession no.
Chromosome	3,868,812	44.18	3,981	83	30	CP082363
Plasmid pPM400	395,960	41.54	366	16	3	CP082364
Plasmid pPM8	8,202	34.86	10			CP082365
Plasmid pPM6	5,458	36.42	5			CP082366
Plasmid pPM5	5,007	36.53	6			CP082367
Plasmid pPM4	3,841	38.35	4			CP082368
Plasmid pPM2	1,768	37.73	2			CP082369
Total	4,289,048		4,374			

### Data availability.

This project has been deposited at GenBank under the accession numbers OK217261 (16S rRNA gene), CP082363 (chromosome), CP082364 to CP082369 (plasmids), BioProject accession number PRJNA759103, BioSample accession number SAMN21156371, and the Sequence Read Archive accession numbers SRR15710034 and SRR16004257 (Illumina and ONT reads, respectively).
